# Nitrogen Addition Enhances Drought Sensitivity of Young Deciduous Tree Species

**DOI:** 10.3389/fpls.2016.01100

**Published:** 2016-07-22

**Authors:** Christoph Dziedek, Werner Härdtle, Goddert von Oheimb, Andreas Fichtner

**Affiliations:** ^1^Institute of Ecology, Leuphana University of LüneburgLüneburg, Germany; ^2^Institute of General Ecology and Environmental Protection, Dresden University of TechnologyTharandt, Germany

**Keywords:** climate change, complementarity, ecosystem functioning, insurance hypothesis, nitrogen deposition, plant–climate interactions, temperate forest, tree growth

## Abstract

Understanding how trees respond to global change drivers is central to predict changes in forest structure and functions. Although there is evidence on the mode of nitrogen (N) and drought (D) effects on tree growth, our understanding of the interplay of these factors is still limited. Simultaneously, as mixtures are expected to be less sensitive to global change as compared to monocultures, we aimed to investigate the combined effects of N addition and D on the productivity of three tree species (*Fagus sylvatica*, *Quercus petraea*, *Pseudotsuga menziesii*) in relation to functional diverse species mixtures using data from a 4-year field experiment in Northwest Germany. Here we show that species mixing can mitigate the negative effects of combined N fertilization and D events, but the community response is mainly driven by the combination of certain traits rather than the tree species richness of a community. For beech, we found that negative effects of D on growth rates were amplified by N fertilization (i.e., combined treatment effects were non-additive), while for oak and fir, the simultaneous effects of N and D were additive. Beech and oak were identified as most sensitive to combined N+D effects with a strong size-dependency observed for beech, suggesting that the negative impact of N+D becomes stronger with time as beech grows larger. As a consequence, the net biodiversity effect declined at the community level, which can be mainly assigned to a distinct loss of complementarity in beech-oak mixtures. This pattern, however, was not evident in the other species-mixtures, indicating that neighborhood composition (i.e., trait combination), but not tree species richness mediated the relationship between tree diversity and treatment effects on tree growth. Our findings point to the importance of the qualitative role (‘trait portfolio’) that biodiversity play in determining resistance of diverse tree communities to environmental changes. As such, they provide further understanding for adaptive management strategies in the context of global change.

## Introduction

Forest ecosystems are currently facing unprecedented shifts in environmental conditions, with implications for biodiversity patterns, ecosystem functions and services (Anderson-Teixeira et al., 2015). Important drivers of environmental shifts are, among others, climate change and atmospheric changes, for example the deposition of reactive forms of nitrogen (Vitousek et al., 1997; Sala et al., 2000). Climate change, accompanied by increasing temperatures and more frequent drought events (IPCC, 2013), is expected to severely affect carbon and water cycles of forest ecosystems (Grossiord et al., 2014). Moreover, drought events and increasing summer temperatures may impose constraints on growth and competitiveness of trees species that are considered sensitive to water shortage (Geßler et al., 2007; Grossiord et al., 2014). On the other hand, atmospheric deposition of nitrogen (N) has tripled since 1860 and is expected to further increase in coming decades (Galloway et al., 2004). In forest ecosystems, N deposition is considered responsible for accelerated biomass increment in recent decades, because tree growth is often limited by the availability of N (Rennenberg et al., 1998; Nadelhoffer, 2000; Pretzsch et al., 2014). Long-term N loading has also been shown to alter soil nutrient cycling and promote soil acidification, leaching of nitrate and soil cations (Magill et al., 1997; Aber et al., 1998; Rennenberg et al., 1998). Consequently, both an increase in nitrogen deposition and drought events may have severe consequences for forest community dynamics, and thus for ecosystem functioning and services.

Due to the global importance of forest ecosystems, there is a bulk of research that addressed the effects of global change drivers on various ecosystem functions (for a global overview see Allen et al., 2010; Bobbink et al., 2010). Many studies, however, have focused on single-factor approaches, whereas analyses on interaction effects are scarce (Zavaleta et al., 2003; Yang et al., 2013), particularly for combined N and D effects (Nilsen, 1995; Meyer-Grünefeldt et al., 2015b,a). It is conceivable, for example, that co-occurring drivers of global change do not act additively (i.e., the summation of single effects), but have non-additive effects on ecosystem responses (i.e., show antagonistic or synergistic interactions; Meyer-Grünefeldt et al., 2015b). This implies that ecosystem responses to multiple environmental shifts cannot be inferred from single-factor studies alone, and emphasizes the need for concomitant multi-factor approaches (Lindenmayer et al., 2010; Ochoa-Hueso et al., 2014; Hautier et al., 2014).

Next to the physiological response of individual trees, the structure and composition of forest ecosystems is central for allowing forest to adapt to global environmental changes (Coomes et al., 2014; De Frenne et al., 2015). In this context, species diversity is assumed to mitigate climate change effects on forest productivity, because diverse forests are expected to react less sensitively to environmental shifts as compared to monocultures (Filotas et al., 2014). Overall, there is increasing evidence that biodiversity promotes various ecosystem functions and services (e.g., Cardinale et al., 2012), and three main mechanisms have been proved to drive diversity-functioning relationships: complementarity (i.e., resource partitioning and facilitation), selection (or sampling) effects (i.e., the higher likelihood that mixtures contain highly productive species) and ecological insurance (Loreau and Hector, 2001; Scherer-Lorenzen, 2014). Many recent biodiversity-ecosystem functioning experiments provided evidence that increasing diversity can reduce the variability of ecosystem properties, and thus increase the temporal stability (e.g., in terms of resistance or resilience) at the ecosystem level (Tilman et al., 2006; Hector et al., 2010; Proulx et al., 2010; Isbell et al., 2015). For instance, observational and simulational studies have shown a positive relationship between tree species richness and the stability of wood production (Jucker et al., 2014; Morin et al., 2014). This beneficial stabilizing effect of biodiversity, also termed as ‘insurance hypothesis’ (Yachi and Loreau, 1999), can arise from overyielding (i.e., the productivity of mixtures is higher than the average of the monocultures or most productive monoculture), the spatial (i.e., niche partitioning), or temporal (i.e., species asynchrony) complementarity between species or facilitative plant-interactions (Loreau, 2010; Hector et al., 2010; McIntire and Fajardo, 2014). Thus, biodiversity related ‘insurance effects’ imply that diverse forests are composed of tree species that (i) differ with regard to intrinsic responses to environmental change, (ii) differ with regard to the speed with which they respond to environmental disturbances, or (iii) show a reduction in the strength of competition (Loreau and de Mazancourt, 2013).

We evaluated how N addition and drought interactively affect tree growth in monocultures and mixtures. In a 4-year field experiment with juvenile trees, in which we altered species combinations and species richness levels, we exposed monocultures and mixtures to full-factorial combinations of summer drought and N fertilization. Experiments were conducted with three different tree species: European beech (*Fagus sylvatica*), Sessile oak (*Quercus petraea*), and Douglas fir (*Pseudotsuga menziesii*), henceforth referred to as beech, oak, and fir, respectively. These species differ in key functional traits that are linked to productivity and shade tolerance (e.g., specific leaf area, leaf longevity, and wood density) and are considered to be ecologically and/or economically important from a European perspective (Valladares and Niinemets, 2008; Ellenberg and Leuschner, 2010; Lasky et al., 2014). Specifically, we asked, how sole and combined effects of N fertilization and drought mediate (i) tree-level growth in relation to species identity (of the target and neighboring trees), (ii) stand-level growth in relation to species combination and richness, and (iii) complementarity and selection effects and thus net biodiversity effects of tree communities.

## Materials and Methods

### Study Area

All experimental sites were established in near-natural broad-leaved forest ecosystems typical of the lowlands of NW Germany (Lower Saxony, 53° 8′ 7.827″ N 10° 22′ 20.96″). Soils of the study area developed from sediments of the penultimate glacial period, and prevailing soil types are acidic Cambisols or Luvisols (according to the WRB system, 2006). Mean pH_H2O_-values in the upper mineral (A-) horizon ranged between 3.9 and 4.7. The natural forest communities at these sites are acidic beech forests that belong to the Galio odorati-Fagetum (Ellenberg and Leuschner, 2010). The climate is of a sub-oceanic type. Mean precipitation is 718 mm yr^-1^, and the annual mean temperature is 9.2°C.

### Experimental Design and Plant Material

In April 2010 we established a 4-year field experiment using a randomized block design (with seven replicate blocks). Blocks were established under larger canopy gaps (0.25–0.50 ha in size) to simulate a quasi-natural regeneration situation under an opened canopy. All blocks were fenced during the experiment to exclude grazing effects. Each block consisted of six plots with different species combinations, where three target species (beech, oak, and fir) were grown, either in monoculture, 2-species or 3-species mixtures (for species combinations see **Table 1**). Each plot was divided into four subplots (1 m × 1 m) with 0.5 m wide buffer strips, and each subplot was randomly assigned to one of the following treatments: control, nitrogen (N) fertilization, drought treatment, and a combination of N fertilization and drought treatment (henceforth referred to as control, N treatment, D treatment, and N+D treatment, respectively). The experiment thus comprised six species combinations and four treatment levels, resulting in a total of 24 experimental combinations (each 7 × replicated).

**Table 1:** Design of the experiment.

Species	Diversity level	Species combination	No. trees
*Fagus sylvatica* (beech)	mono	–	252
*Quercus petraea* (oak)	mono	–	252
*Pseudotsuga menziesii* (fir)	mono	–	252
*Fagus sylvatica*	mix2	beech-oak	140
*Fagus sylvatica*	mix2	beech-fir	140
*Quercus petraea*	mix2	beech-oak	112
*Pseudotsuga menziesii*	mix2	beech-fir	112
*Fagus sylvatica*	mix3	beech-oak-fir	84
*Quercus petraea*	mix3	beech-oak-fir	84
*Pseudotsuga menziesii*	mix3	beech-oak-fir	84

Total			1512

*Number of planted target trees of each diversity level and species combination. Mono, monoculture; mix2, 2-species mixture; mix3, 3-species mixture.*

In April 2010, each subplot was planted with 25 3-year-old tree saplings (planting distance: 20 cm), which originated from a local forest nursery. In mixed-species subplots, trees were planted in a systematic species alteration pattern (e.g., beech-oak-fir-beech-oak-fir etc.). To account for edge effects, only the central nine individuals were considered as target trees for subsequent analyses. All treatments started in the year 2012, i.e., 2 years after sapling planting. This delayed start was chosen to avoid confounding effects between experimental treatments and planting.

In the N treatments (i.e., N and N+D), N was applied (as NH_4_NO_3_) in a quantity equivalent to 50 kg N ha^-1^ yr^-1^ (as solution in deionized water). This treatment strength was chosen to simulate the effects of atmospheric N deposition which some forest ecosystems currently receive in NW Europe (with 50 kg N ha^-1^ yr^-1^ representing the upper range limit of current deposition rates; Galloway et al., 2004; Bobbink et al., 2010).

To simulate summer drought events (D treatments; i.e., D and N+D) we installed rain-out shelters (2–3 m aboveground) with UV transparent foil (UV-B Window, folitec GmbH, Westerburg, Germany) in the respective subplots to exclude any precipitation. The rain-out shelters were installed from July 9th to July 31st and August 13th to September 7th in 2012, and from July 5th to September 5th in 2013. Effects of D treatments on soil water contents were determined by means of volumetric soil water content sensors (based on Time Domain Reflectometry; Decagon Devices, Pullman, WA, USA) that were installed in four representative blocks in 2012 and 2013 in the upper mineral soil (0–5 cm). Measurements of volumetric soil water contents indicated that D treatments reduced the soil water content by about 20% (volumetric losses compared to field capacity, achieved during the last week of the D treatments), which corresponds to a moderate-severe drought event in the study region (Rose et al., 2009).

### Tree Measurements

For all trees, height and biomass were determined. Tree height (measured from the root collar to the top) was recorded at the beginning of the treatment application (April 2012) and at the end of the experiment in September 2013, which corresponded to a 2-year growing period. For each tree we calculated relative growth rate (RGR) of tree height as RGR = (ln H_2_ – ln H_1_)/(t_2_ – t_1_), where H_1_ and H_2_ are the tree heights at the beginning (t_1_) and end (t_2_) of the experiment. We used RGR instead of absolute growth rates as a response variable to model individual tree growth, because RGR is less sensitive toward differences in initial size (Mencuccini et al., 2005). After tree harvest (September 2013), we additionally measured the stem biomass (including branches) and the biomass of leaves or needles for all tree individuals. Biomass samples were dried at 40°C for 3 days (until weight constancy) and subsequently weighted. Target tree characteristics are provided in **Table 2**.

**Table 2:** Target tree characteristics of the three study species.

	Mean (*SD*)	Range
***Fagus sylvatica***		
Initial tree height (cm)	89.4 (16.5)	52.0–141.0
AGB (g)	51.5 (41.4)	4.5–305.1
AGR (cm year^-1^)	16.7 (11.4)	0.0–55.5
RGR (cm cm^-1^ year^-1^)	0.15 (0.08)	0.0–0.37
***Quercus petraea***		
Initial tree height (cm)	101.0 (25.2)	38.0–178.0
AGB (g)	53.0 (48.3)	0.1–323.8
AGR (cm year^-1^)	17.2 (12.3)	0.0–59.0
RGR (cm cm^-1^ year^-1^)	0.13 (0.08)	0.0–0.36
***Pseudotsuga menziesii***		
Initial tree height (cm)	118.6 (25.9)	62.0–202.0
AGB (g)	150.3 (107.7)	16.2–683.2
AGR (cm year^-1^)	28.6 (13.7)	0.0–87.0
RGR (cm cm^-1^ year^-1^)	0.19 (0.07)	0.0–0.37


*Absolute (AGR) and relative growth rate (RGR) of tree height refer to a 2-year census interval; AGB, aboveground biomass at the end of the census interval.*

### Data Analysis

Individual tree growth analyses was focused on 1291 target trees in total (beech: 558, oak: 320, fir: 413). Due to mortality, 12% of the original 1512 target trees were not available to be measured at the end of the experiment. Oak showed highest mortality, followed by fir and beech, but we found no statistically significant treatment effect across species (beech: *P* = 0.10; oak: *P* = 0.91; fir: *P* = 0.83; **Supplementary Figure S1**). Moreover, observations with negative growth rates (3% of the surviving trees) were assumed to be damage-related (e.g., due to planting failures or falling large-sized branches) or to have measurement error, and therefore omitted in the subsequent height growth analysis to avoid biased estimates. However, trees with zero increments were retained.

To examine the tree size, treatment, and species diversity (measured as species richness) dependence of RGR of the three target species, we applied linear mixed models using block, plot and treatment as nested random factors. We fitted several alternative models for each target species separately including initial height, treatment, species combination, and their interactions as fixed effects. To address the skewed response and heteroscedasticity of the beech and oak growth data, the residual error was modeled using a variance function based on the power of the fitted values (Pinheiro and Bates, 2004). Models were selected based on the Akaike Information Criterion (AIC) and maximum likelihood (ML) estimations. Moreover, we ranked the models based on Akaike weights (*w_i_*), which are the relative likelihood of each model to be the best-fitting model, given the complete set of candidate models (Burnham and Anderson, 2002). Only models with an AIC difference (ΔAIC) ≤ 2 (compared with the best-fitting model) were considered as models with substantial support (Burnham and Anderson, 2002), and for each species the model with the highest Akaike weights was chosen as the most parsimonious model. Parameter estimates of the best-fitting models were based on the restricted maximum likelihood (REML) method.

The strength of each treatment effect on RGR rates was determined by the magnitude of treatment effect (MTE). MTE was calculated as MTE = (X_T_ – X_C_)/(X_T_ + X_C_), where X_T_ is the predicted response of target tree *i* in the global change driver treatments (N, D, N+D) and X_C_ the predicted response in the control (C) treatment. This index ranges from –1 (negative global change driver influence) to +1 (positive global change driver influence) for each species, thus facilitating between-species comparisons. Differences in MTE among species were evaluated by analysis of variance (ANOVA) with a *post hoc* performance (Tukey HSD test).

Total aboveground biomass (all woody compartments and leaves; AGB) was used as a response for tree vigor. For trees that died during the experiment we used the average species-specific AGB of each treatment and species combination. We applied the additive partitioning method according to Loreau and Hector (2001) to quantify the net biodiversity effect (NE) on AGB of species mixtures, which we further partitioned into the complementarity (CE), and selection effect (SE). NE, CE, and SE were calculated using the following equations:

$$NE=\Sigma Y-\overset{¯}{M}$$

$$CE=N\times \overset{¯}{M}\times \overset{¯}{\Delta RY}$$

SE=N×cov⁡(M, ΔRY)

where Y is the observed AGB for each species in mixture and M is the yield of a species growing in monoculture. N is the number of species and ΔRY the deviation from the expected relative yield of a species in mixture (ΔRY = (Y/M) – (1/N)).

To account for size differences of the species-mixtures, and thus allow for inter-site comparisons, diversity components were standardized dividing NE, CE, and SE by the expected AGB based on monocultures (see Morin et al., 2011). For the subsequent analysis these values were square-root transformed to meet the model assumptions while preserving the original positive and negative signs (Loreau and Hector, 2001). For each species combination we fitted a linear-mixed effects model using treatment as fixed effect and block as random factor to account for potential differences in site conditions. All statistical analyses were performed in R (version 3.1.0^1^) using the packages *nlme* and *MuMIn*.

## Results

### Effects of Nitrogen Fertilization and Drought on Tree-Level Height Growth

For all species the minimum adequate models according to the AIC included tree size, treatment and species composition effects (**Table 3**). For beech, the treatment effects significantly depended on tree size (*P* < 0.01; **Table 4**), with treatment effects becoming more pronounced with increasing height. For oak and fir, the RGR-treatment relationships were consistent across the observed height range. Compared to control plots, RGR of oak was significantly lower in the N+D treatment (*P* < 0.05), and marginally significant lower in the N treatment (*P* ≤ 0.1), while a significant decline in RGR of fir was induced by drought (*P* < 0.05). Moreover, a significant species composition effect on the shape of the size response was observed for beech (*P* < 0.001) and oak (*P* < 0.01), while for fir, the species composition effect (*P* < 0.01) was independent of tree size (**Table 4**). There was no support for a statistically significant three-way interaction effect on RGR, showing that for each species the size-treatment relationship did not shift with species composition (**Table 3**). Graphical validation plots indicated unbiased estimates. The best-supported models explained between 41% (beech), 44% (oak), and 51% (fir) of the variation in RGR of height.

**Table 3:** Model selection statistics (Akaike Information Criterion ΔAIC and Akaike weights *w*_i_) for various candidate models describing the RGR of tree height as a function of initial tree height (H), treatment (T), and species composition (C) effects of European beech (*Fagus sylvatica*), Sessile oak (*Quercus petraea*), and Douglas fir (*Pseudotsuga menziesii*).

Model	Fixed effects							*Fagus sylvatica*		*Quercus petraea*		*Pseudotsuga menziesii*	
				
	H	T	C	H × T	H × C	T × C	H × T × C	ΔAIC	*w*_i_	ΔAIC	*w*_i_	ΔAIC	*w*_i_
1	×	×						23.99	0.00	4.47	0.08	11.57	0.00
2	×		×					15.72	0.00	7.94	0.01	2.38	0.09
3		×	×					73.76	0.00	9.46	0.01	7.21	0.01
4	×	×	×					19.45	0.00	7.25	0.02	**0.01**	**0.30**
5	×	×	×	×				13.67	0.00	10.08	0.00	0.00	0.30
6	×	×	×		×			10.82	0.00	**0.13**	**0.50**	1.20	0.16
7	×	×	×			×		25.29	0.00	8.32	0.01	7.38	0.01
8	×	×	×	×	×			**0.00**	**0.91**	3.51	0.07	1.75	0.12
9	×	×	×	×		×		19.64	0.00	8.55	0.00	7.81	0.01
10	×	×	×		×	×		14.00	0.00	0.00	0.26	8.42	0.00
11	×	×	×	×	×	x		4.74	0.08	2.55	0.05	9.46	0.00
12	×	×	×	×	×	×	x	15.61	0.00	5.20	0.00	15.21	0.00


*The best-supported models with the highest Akaike weights are highlighted in bold. For Douglas fir the more parsimonious model that included a marginal significant height-treatment interaction (*P* = 0.09) was rejected, since the main effects-only model fit the data equally well (ΔAIC = 0.01, *w*_i_ for both models = 30%).*

**Table 4:** Best-fitting mixed-effects models for RGR of tree height of (a) European beech (*Fagus sylvatica*), (b) Sessile oak (*Quercus petraea*) and (c) Douglas fir (*Pseudotsuga menziesii*).

Fixed effects	d.f.	*L*-ratio	*P*-value
**(a) *Fagus sylvatica***			
Initial tree height (H)	1	56.3	<0.001
Treatment (T)	3	2.3	0.517
Species composition (C)	3	10.5	0.014
H × T	3	15.3	0.002
H × C	3	20.9	<0.001
**(b) *Quercus petraea***			
Initial tree height (H)	1	4.2	0.040
Treatment (T)	3	6.7	0.082
Species composition (C)	2	1.2	0.543
H × C	2	10.0	0.007
**(c) *Pseudotsuga menziesii***	
Initial tree height (H)	1	15.6	<0.001
Treatment (T)	3	8.4	0.039
Species composition (C)	2	9.2	0.002


**P*-values were derived from likelihood-ratio tests based on maximum likelihood (ML) estimations.*

**FIGURE 1:**
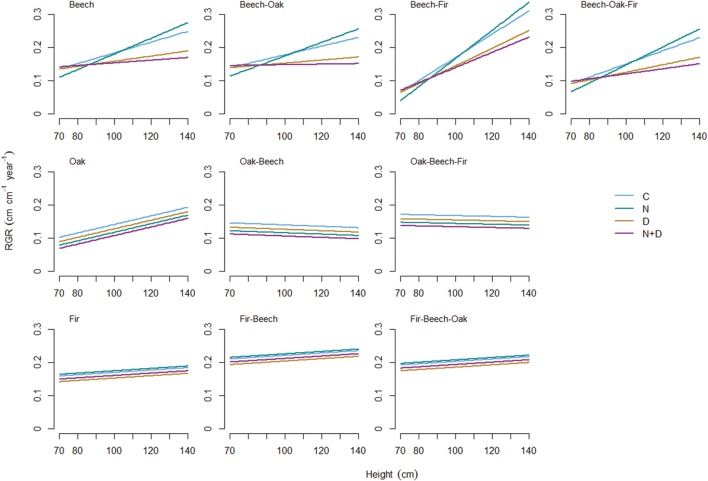
**Relationship between tree size (initial height), global change effects (C, control; N, nitrogen addition; D, drought; N+D: nitrogen addition plus drought), species diversity (monoculture; 2-species mixture, and 3-species mixture) and relative growth rate (RGR) of tree height for European beech (*Fagus sylvatica;* upper row), Sessile oak (*Quercus petraea;* middle row) and Douglas fir (*Pseudotsuga menziesii*; lower row).** Regression lines are based on the predictions of the best-fitted models in **Table 4**.

**FIGURE 2:**
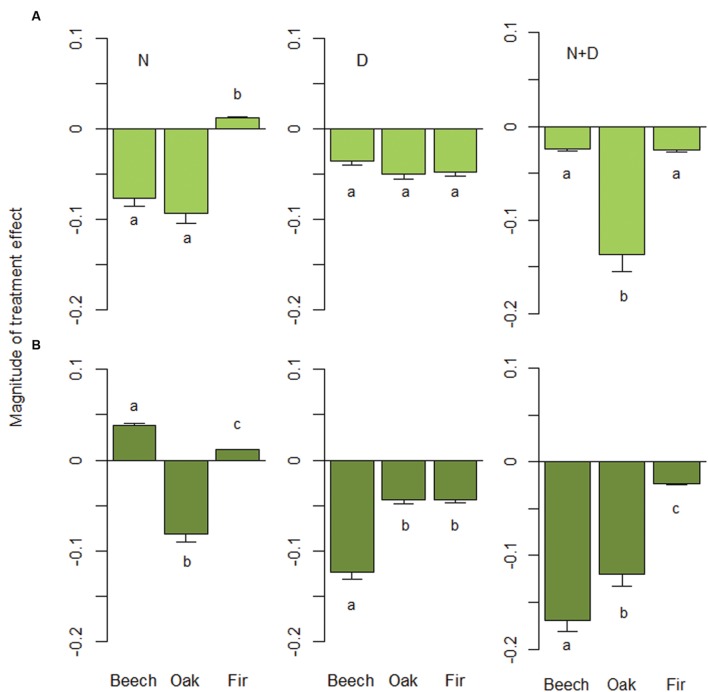
**Magnitude of treatment effect (MTE) for nitrogen addition (N), drought (D) and their combination (N+D).** For each species (European beech: *Fagus sylvatica*, Sessile oak: *Quercus petraea*, and Douglas fir: *Pseudotsuga menziesii*), MTEs were predicted for **(A)** small-sized (initial height of 80 cm) and **(B)** large-sized trees (initial height of 130 cm) based on our best-fitting models (see **Table 4**). Error bars show standard errors based on across-species combination response. Different letters indicate significant differences (Tukey-test: *P* < 0.05) among species.

The positive RGR-size relationship was most pronounced for beech with a greater increase in growth rates when growing in mixture with fir (**Figure 1**). Similarly, RGR of oak trees in monoculture increased with size. In contrast, the influence of size was less evident for oak growing in 2- or 3-species mixtures and fir growing either in monoculture or mixture. The mode of growth response to treatment effects, however, was significantly different among species and tree sizes (**Figure 2**). Oak and fir showed an additive response (summation of the single effects) to simultaneous N addition and drought, whereas the response of beech was non-additive (i.e., an antagonistic response of smaller and a synergistic response of larger individuals). This trend was consistent along the investigated diversity gradient, since we did not observe interacting effects of treatment and species composition (**Table 3**).

Compared to the control, RGR of small trees in the N trea- tment was lower for beech and oak, but higher for fir (**Figure 2**). In contrast to oak, growth rates of large beech and fir trees were enhanced by nitrogen enrichment (*P*_adj_*_._* < 0.01). In contrast, drought reduced height growth of all species and sizes with effects being strongest for large-sized beech trees (*P*_adj._ < 0.001). The combination of N addition and drought was negative for all species, but size-dependency was strongest for beech. The sensitivity of oak and fir to N+D was equally high for small and large trees, with effects being much stronger for oak. Large beech trees, however, suffered most from N+D, resulting in a sevenfold decline in growth rates compared to small individuals. Thus, growth reductions induced by combined N and D effects of large individuals significantly increased within the series fir < oak < beech (all comparisons: *P*_adj._ < 0.05; **Figure 2**).

AGB was closely related to RGR, and the strength of the relationship did not significantly differ among species (**Supplementary Figure S2**).

### Effects of Nitrogen Fertilization and Drought on Stand-Level Biomass Production

**FIGURE 3:**
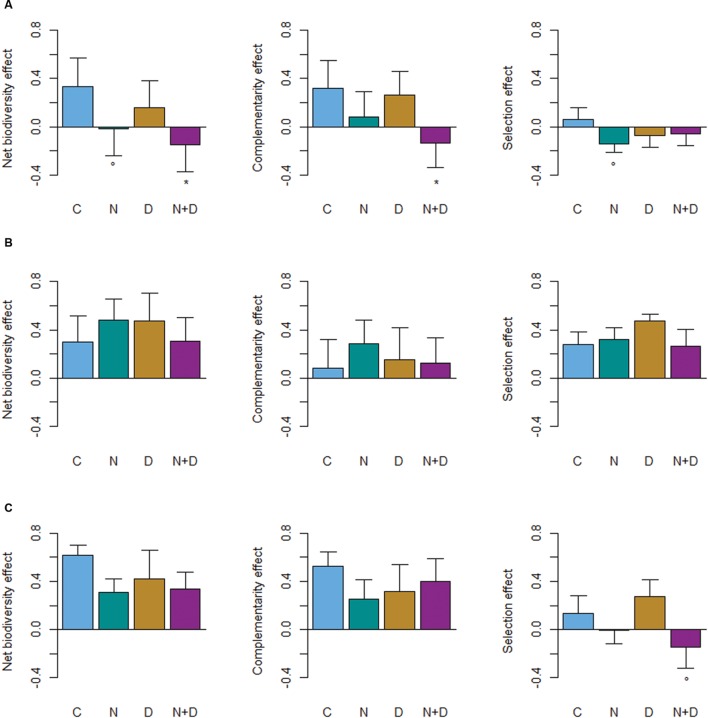
**Changes in net biodiversity, complementarity and selection effects with global change drivers (C, control; N, nitrogen addition; D, drought; N+D: nitrogen addition and drought) for **(A)** beech-oak stands (*Fagus sylvatica*–*Quercus petraea*), **(B)** beech-fir stands (*Fagus sylvatica*–*Pseudotsuga menziesii*), and **(C)** beech-oak- fir stands (*Fagus sylvatica*–*Quercus petraea*–*Pseudotsuga menziesii*).** Values are square-root transformed to meet model assumptions while preserving the original positive and negative signs. Asterisks indicate significant differences (*P* < 0.05) and open circles marginal significant differences (*P* ≤ 0.1) between the control (C) plots and global change driver (N, D, N+D) treatments.

In the absence of D or N treatments the mixture effect on overall stand productivity was positive for all species mixtures (**Figure 3**). Overyielding was evident in 81% of the control plots and in 69% of the sites (blocks) across treatments (**Supplementary Table S1**), but we observed a large variation across sites (**Supplementary Table S2**).

The impact of global change drivers (D, N, or N+D) on the net biodiversity effect was driven by species identity rather than species diversity. Regardless of treatment, the average net diversity effects of beech-fir and beech-oak-fir stands remained positive and did not statistically differ from the control (**Figure 3**). In contrast, for beech-oak mixtures the magnitude and direction of diversity effects differed between treatments. N addition reduced the positive effect of species mixture to become neutral (*P* = 0.1), whereas the combined effects of N addition and drought caused a significant loss of biodiversity effects and underyielding, respectively (*P* < 0.05; **Figure 3**). This pattern can be primarily attributed to the loss of complementarity effects with regard to the N+D treatment (*P* < 0.05) and selection effects in relation to the N treatment (*P* < 0.1). Similarly, different underlying complementarity and selection effects were obvious for beech-fir and beech-oak-fir mixtures. In 61% of the beech-fir sites the selection effect was greater than the complementarity effect, particularly in the D treatment. Thus, high stand biomass productivities can be mainly ascribed to fir. In the 3-species mixture the selection effect became negative in the N+D treatment (*P* = 0.1) and neutral in the N treatment, but CE were always greater than SE (**Figure 3**).

## Discussion

### Species-Specific Growth Response to Combined Effects of Nitrogen Addition and Drought

Our results show that tree growth response to treatments was mainly driven by species identity rather than species diversity, and the combined effects of N and D treatments proved to be both additive and non-additive. In the first case, the combined effects of N+D on RGR of tree height corresponded with the sum of the single effects (oak and fir), but in the latter case the combination of both factors caused negative growth responses, with mutually amplifying effects (for large beech trees, despite the positive single effect of N fertilization). This finding suggests that – at least in the case of beech – growth responses to environmental shifts are difficult to infer from species responses to single factors (Zavaleta et al., 2003). Several mechanisms may account for the non-additive effects of N+D treatments. First, N fertilization often results in a shift in biomass allocation patterns (in favor of aboveground biomass), resulting in a concomitant increase of biomass shoot-root ratios (Thomas and Hilker, 2000; Meyer-Grünefeldt et al., 2015b). For example, leave biomass investments of coniferous tree species increased with N fertilization (Högberg et al., 1993), and can thus increase the water consumption and probability of water stress (Nilsen, 1995). The responses described above are in agreement with the ‘resource optimization hypothesis’, according to which plants show (relatively) higher aboveground investments (and hence higher shoot-root ratios) with increasing nutrient availability (McConnaughay and Coleman, 1999; Ågren and Franklin, 2003). High shoot-root ratios, in turn, can lead to increasing evaporative demands and thus a higher sensitivity to drought events (Meyer-Grünefeldt et al., 2015b). Second, N fertilization can increase fine- and coarse-root mortality and decrease the mycorrhiza colonization, both of which can impair supply and therefore increase their drought sensitivity (Hendricks et al., 2000; Nadelhoffer, 2000; Teste et al., 2012). Third, as trees can optimize the fine root and branch hydraulic system in water-limited environments (Hertel et al., 2013; Schuldt et al., 2016), an increasing N availability might prevent such adaptation mechanisms and therefore increase the suspectibility to drougth.

Tree species also responded differently to N fertilization, with a facilitation of (large) beech and fir trees, but adverse effects on oak. Deleterious effects of N fertilization on juvenile oak trees have also been reported in the study of BassiriRad et al. (2015), without a clear indication of the underlying mechanisms. In our study, species-specific responses are likely related to their traits and competitive hierarchy. Oak trees are light-demanding and may suffer from an unfavorable light environment when overgrown from larger neighbors, particularly at N-fertilized sites (Ellenberg and Leuschner, 2010). In this context, the strong size-asymmetry of treatment effects for beech suggests that our findings are related to size-asymmetric competition, because larger individuals mostly obtain a disproportionate share of resources and thus suppress the growth of smaller individuals (Potvin and Dutilleul, 2009). As a consequence, larger trees have a competitive advantage in resource acquisition over smaller individuals, and thus benefit most from additional nutrients, explaining the N-induced height growth decline of smaller oak and beech trees.

Species differences in the sensitivity to drought, as shown for larger individuals in our study, coincides with the well-known ecophysiology of these species (see for example Thomas, 2000; Geßler et al., 2007; Meier and Leuschner, 2008; Friedrichs et al., 2009; Härdtle et al., 2014). In a study of five temperate adult tree species, Zimmermann et al. (2015) found that beech is most susceptible to drought, which is in line with our observed increasing drought sensitivity as beech trees grew larger. Thus, species-specific differences in drought sensitivity might result in shifts in the competitive hierarchy in mixed-species tree communities. Our study, however, provided no evidence for changes of treatment effects depending on community composition. This suggests that treatment effects at the scale of individual trees were highly species-specific, and growth responses of juvenile trees to treatments were strongly mediated by the species’ trait characteristics (also see discussion below) and local neigborhood conditions (Lübbe et al., 2015, 2016). An additional explanation to the statistically non-significant three-way interaction (H × T × C) and two-way interaction (T × C) is that diversity effects may need time to fully evolve in long-living plant communities such as forests, and therefore may become more pronounced as trees become larger.

We found that tree size-related changes in RGR were context-specific (neighborhood composition) and varied with species identity. Species interactions leading to a spatial complementarity in resource use due to differences in leaf habit (e.g., Coomes et al., 2009) are likely to be important in beech-fir mixtures. As a result, species mixing can mitigate drought susceptibility of mature beech trees by reducing intra-specific competition (Metz et al., 2016). In contrast, oak trees (as the most light-demanding species) proved to be week competitors (at least under the given experimental settings), and benefitted most from growing with conspecific neighbors. Thus, positive mixture effects in our study may be primarily the result of trait induced competitive hierarchies (Kunstler et al., 2012) and the species’ trait characteristics also accounted for the observed interacting effects of tree size and species composition.

### Functional Composition of Forests Modulate the Effects of Nitrogen Addition and Drought on Stand Productivity

Overyielding was evident for almost all plots across treatments, which is in agreement with many previous studies reporting a positive effect of tree diversity on forest productivity (e.g., Paquette and Messier, 2011; Vilà et al., 2013; Forrester and Bauhus, 2016). However, in our experiment the NE on stand-level productivity strongly depended on both the species composition and the species-specific responses to treatments. In the beech-oak mixture, we found a significant underyielding in the N+D treatment, attributable to negative N+D effects on CE. We hypothesize that the negative NE was brought about by the negative responses of beech and oak to N+D treatments already observed at the tree-level. This, in turn, would indicate that stand-level, and tree-level responses to ‘environmental shifts’ are closely related, or, more specifically, may depend on the trait characteristics of the species included in a mixture (Lübbe et al., 2015). This interpretation is supported by the result that we found no NE and a negative SE for beech-oak mixtures in the N treatment, likely brought about by the strong negative response of oak trees to N fertilization. We conclude that the resistance of a species mixture to environmental shifts may be more determined by the traits typical of the species included in a mixture than by the mere complementarity of the traits (or the functional dissimilarity) of these species (as given in the case of beech and oak). Biodiversity thus would not serve *per se* as an ‘insurance’ for the mitigation of global change effects on ecosystem functions (Lübbe et al., 2015), but would act in terms of a ‘trait portfolio’ that preserves a broad spectrum of functional traits enabling a species’ resistance to environmental stressor (comparable to a lock-and-key model, according to which only particular traits ensure higher resistance of plant communities to environmental shifts; Polley et al., 2013). This perspective emphasizes the importance of both the quantity and quality of biodiversity for ecosystem resistance to environmental change (Mouillot et al., 2013).

The hypothesis provided above also supports the interpre- tation of treatment responses of those mixtures in which fir was included (i.e., beech-fir and beech-oak-fir mixtures). In these mixtures we found positive NE across treatments, suggesting that fir acted as a kind of ‘buffer’ mitigating the (partly negative) effects of N fertilization and drought. In the beech-fir mixture, positive NE were mainly attributable to SE, particularly in the D treatment. Obviously, the low sensitivity of fir to D and N+D treatments (of small and small + large trees, respectively; see Bansal et al., 2015) was conveyed to the stand-level, resulting in the observed positive NE across treatments. In the 3-species mixture, fir obviously mitigated the adverse effects of N and N+D observed for the beech-oak mixture, resulting in positive CE (substantially contributing to the NE). We hypothesize that trait-characteristics of fir mainly concurred to the observed response pattern (e.g., its low drought sensitivity; Bansal et al., 2015), resulting in an increased stand-level resistance of the tree-mixture. In summary, stand-level responses to treatments (and corresponding NE) were strongly mediated by species composition and the species’ functional trait characteristics included in a mixture. This finding is in line with our observation on the individual tree level and matches observations in other tree diversity experiments, according to which species identity often proved to be as influential as species richness effects on productivity patterns (Jacob et al., 2010; Lang et al., 2012; Grossiord et al., 2013; Ratcliffe et al., 2015).

## Conclusion

Our results highlight the importance of assessing interacting effects of nitrogen addition and drought to evaluate forest productivity in response to global environmental change. We are aware of the limitation to generalize results from juvenile tree field-experiments to adult tree communities, but manipulations of N and D treatments are hardly achievable in later forest development stages due to the longevity of trees. Hence, our experimental framework provides a unique opportunity to enhance our mechanistic understanding of tree growth in the context of global change by disentangling the effects of various global change drivers and their interactions unequivocally.

We found evidence that the magnitude and direction of combined global change driver effects depend on species identity and neighborhood composition (i.e., trait combination) rather than the level of tree species richness. Thus, species diversity might not mitigate *per se* the impact of drought and increasing N deposition in long-living plant communities. Instead, the occurrence of certain trait combinations (‘trait portfolio’) in diverse communities might act as an ‘insurance’ for the mitigation of global change effects on ecosystem functions. This suggests that the quality of trait composition (‘lock-and-key principle’) is a main component of the ecological insurance hypothesis.

## Author Contributions

WH and GvO conceived the study. CD performed the field and laboratory work, and AF analyzed the data. AF, CD, WH, and GvO wrote the manuscript.

## Conflict of Interest Statement

The authors declare that the research was conducted in the absence of any commercial or financial relationships that could be construed as a potential conflict of interest.

## Footnotes

1 http://www.R-project.org
