# Supplementary material for: Nitrogen Addition Enhances Drought Sensitivity of Young Deciduous Tree Species
Source: Front Plant Sci. 2016 Jul 22;7:1100. doi: 10.3389/fpls.2016.01100 (PMC4957528; doi:10.3389/fpls.2016.01100)

**Supplementary Material**

**Table S1** Treatment specific overyielding, complementarity (CE) and selection (SE) effects for each species mixture.

|  |  |  |  |  |  |  |
| --- | --- | --- | --- | --- | --- | --- |
|  |  |  |  |  |  |  |
| Treatment |  | n (sites) |  | % overyielding |  | % SE > CE |
|  |  |  |  |  |  |  |
|  |  |  |  |  |  |  |
| *Beech-Oak* |  |  |  |  |  |  |
| C |  | 7 |  | 71.4 |  | 14.3 |
| N |  | 7 |  | 42.9 |  | 0.0 |
| D |  | 7 |  | 57.1 |  | 14.3 |
| N+D |  | 7 |  | 28.6 |  | 28.6 |
| Mean |  |  |  | 50.0 |  | 14.3 |
|  |  |  |  |  |  |  |
| *Beech-Fir* |  |  |  |  |  |  |
| C |  | 7 |  | 57.1 |  | 71.4 |
| N |  | 7 |  | 85.7 |  | 57.1 |
| D |  | 7 |  | 71.4 |  | 57.1 |
| N+D |  | 7 |  | 71.4 |  | 57.1 |
| Mean |  |  |  | 75.0 |  | 60.7 |
|  |  |  |  |  |  |  |
| *Beech-Oak-Fir* |  |  |  |  |  |  |
| C |  | 7 |  | 100.0 |  | 14.3 |
| N |  | 7 |  | 71.4 |  | 28.6 |
| D |  | 7 |  | 85.7 |  | 28.6 |
| N+D |  | 7 |  | 85.7 |  | 14.3 |
| Mean |  |  |  | 85.7 |  | 21.4 |
|  |  |  |  |  |  |  |
| Mean (all stands) | | 84 |  | 69.0 |  | 32.1 |
|  |  |  |  |  |  |  |

**Table S2** Intraclass correlation coefficients for the site (block) effect based on biomass production models for each diversity effect-species mixture combination. NE: net biodiversity effect; CE: complementarity effect; SE: selection effect.

|  |  |  |  |  |  |
| --- | --- | --- | --- | --- | --- |
|  |  |  |  |  |  |
| Effect | Beech-Oak |  | Beech-Fir |  | Beech-Oak-Fir |
|  |  |  |  |  |  |
|  |  |  |  |  |  |
| NE | 0.55 |  | 0.03 |  | 0.36 |
| CE | 0.57 |  | 0.04 |  | 0.21 |
| SE | 0.28 |  | 0.00 |  | 0.35 |
|  |  |  |  |  |  |
|  |  |  |  |  |  |

**Figure S1** Treatment-specific observed mortality for (a) European beech (*Fagus sylvatica*), (b) Sessile oak (*Quercus petraea*) and (c) Douglas fir (*Pseudotsuga menziesii*). Across all species, the effect of treatment on mortality rates was not significant (*P* > 0.05) as indicated by generalized mixed effect models (GLMMs with a logit-link function and binomial distribution using block, plot and treatment as nested random factors).


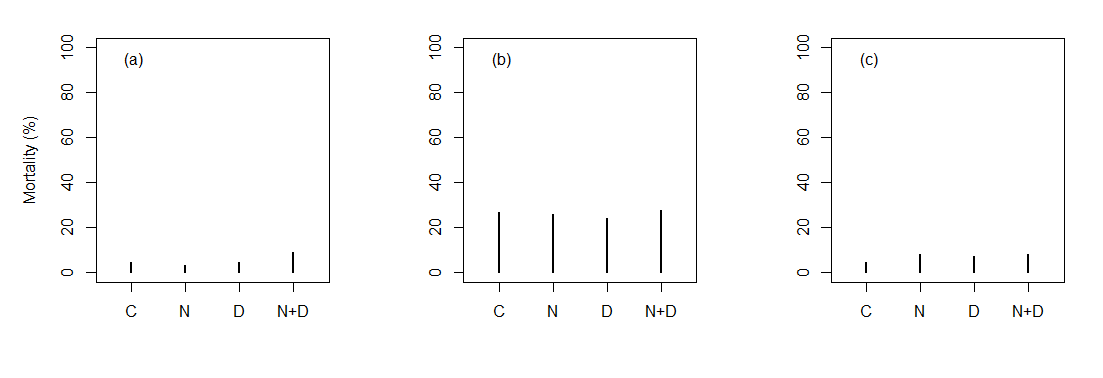


**Figure S2** Relationship between aboveground biomass (AGB) and relative growth rate (RGR) of tree height for (a) European beech (*Fagus sylvatica*), (b) Sessile oak (*Quercus petraea*) and (c) Douglas fir (*Pseudotsuga menziesii*). Grey areas denote the 95% confidence intervals. Panel (d) shows variation in the strength of the AGB-RGR relationship (standardized slope with 95% confidence interval) with species.


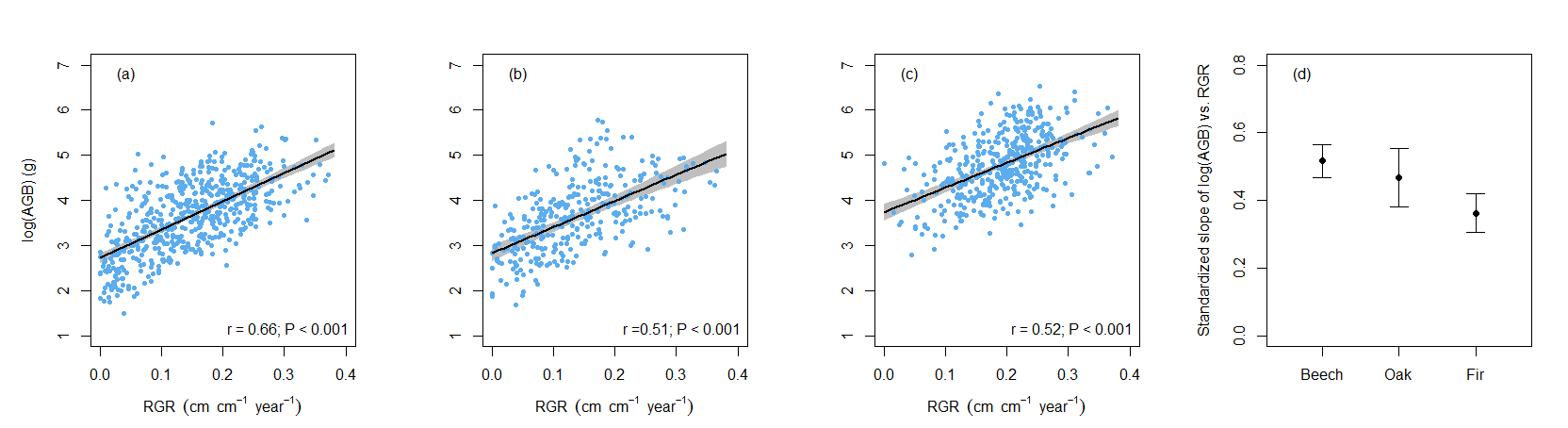

Supplement: Supplementary file 1 [file Data_Sheet_1.DOCX]
